# Filling the Treatment Gap: Geographic Expansion of Buprenorphine Providers Across the U.S.

**DOI:** 10.1016/j.focus.2024.100284

**Published:** 2024-10-16

**Authors:** Suparna Das, Kasimu Muhetaer, Neeraj Gandotra, Naomi Tomoyasu

**Affiliations:** 1 Office of Treatment Services (OTS), Center for Behavioral Health Statistics and Quality (CBHSQ), Substance Abuse Mental Health Services Administration (SAMHSA), Rockville, Maryland; 2 Office of Director (OD), Center for Behavioral Health Statistics and Quality (CBHSQ), Substance Abuse Mental Health Services Administration (SAMHSA), Rockville, Maryland; 3 Office of Chief Medical Officer, Office of Assistant Secretary (OAS), Substance Abuse Mental Health Services Administration (SAMHSA), Rockville, Maryland

**Keywords:** Buprenorphine providers, spatial analysis, access to treatment, X-waiver, MOUD expansion, SAMHSA

## Abstract

**Introduction:**

With opioid-related deaths reaching epidemic levels, gaining a better understanding of access to treatment for opioid use disorder is critical. Buprenorphine is an effective medication for the treatment of opioid addiction. The analysis is critical post–X-waiver elimination, which extended the ability to prescribe buprenorphine for the treatment of opioid use disorder to all practitioners with Drug Enforcement Agency Schedules II–V on their Drug Enforcement Agency registration. The primary purpose of the analysis was to explore the geographic patterns of substance use treatment and establish a geographic baseline to assess the removal of the X-waiver in the future.

**Methods:**

The authors assessed the expansion of buprenorphine providers across the data from all U.S. counties up to December 31, 2022. The authors used all certified buprenorphine providers' data from the database of the buprenorphine waiver notification system. The authors used county-level population data from the U.S. Census of American Community Survey of 2021 and the Centers for Disease Prevention and Control's drug-related mortality data. The authors implemented spatial scan statistics to identify the spatial clusters using SaTScan.

**Results:**

The results from this analysis show that Doctor of Medicine/Doctor of Osteopathic Medicine have the highest numbers of certified providers at 8,134 (65.08%) in 2018 and 14,525 (57.87%) in 2022. This analysis shows that the distribution of buprenorphine providers across the counties of the U.S. was significantly clustered. Higher clusters with RR >1 (*p*<0.001) were found in the states of Washington, Oregon, and Northern California and the Western borders of Montana. Similar clusters of counties with RR >1 (*p*<0.001) were found in the northeastern states of Maine, Vermont, and New Hampshire.

**Conclusions:**

From this analysis, it is evident that buprenorphine-certified providers are clustered in areas of higher drug-related mortality filling a treatment gap. The elimination of the X-waiver will be a significant step toward increasing access to medication for opioid use disorder, and this analysis may be used as a geographic baseline to assess in the future.

## HIGHLIGHTS

• Buprenorphine providers, especially nurse practitioners, have increased yearly since 2018.
• Buprenorphine providers are spatially clustered primarily in the northern counties of the U.S., both on the east and west coasts.
• On the basis of drug-related mortality rates, the spatial clusters were higher in the counties identified as geographic areas of need.

## INTRODUCTION

Preliminary estimates from the Centers for Disease Control and Prevention (CDC) reported 100,306 drug overdose deaths in the U.S. over 12 months ending in April 2021, which showed an increase of 28.5% from the year before.^1^ Among people aged ≥12 years in 2020, 1.0% (or 2.7 million people) had an opioid use disorder (OUD) in the past year.^2^ Medications for OUD (MOUDs), including buprenorphine, methadone, and extended-release naltrexone, were found to be successful treatments that reduce overdose mortality and the other harmful health consequences of OUD.^3^

Of the MOUDs, buprenorphine is a U.S. Food and Drug Administration–approved drug that is considered an essential and cost-effective treatment associated with lowering opioid use and opioid-related mortality.^4^ Buprenorphine, a Schedule III medication under the Controlled Substances Act of 1970, is closely monitored, and the Drug Addiction Treatment Act of 2000 mandated providers prescribing buprenorphine to be either certified addiction specialists or have obtained an X waiver through additional training.^5^^,^^6^ Subsequent revisions have allowed other advanced nonphysician providers, such as nurse practitioners (NPs) and physician assistants, also to be waivered to prescribe buprenorphine.^7^

On April 21, 2021, the HHS released a new Buprenorphine Practice Guidelines, Expanding Access to Treatment for Opioid Use Disorder by removing the longtime requirement tied to training, which some practitioners had cited as a barrier to treating more people. In December 2022, Section 1262 of the Consolidated Appropriations Act, 2023 (also known as Omnibus Bill) removed the federal requirement for practitioners to submit a Notice of Intent (NOI) (waiver) to prescribe buprenorphine for the treatment of OUD. With this provision, Substance Abuse Mental Health Services Administration (SAMHSA) no longer accepts waiver applications. All practitioners who have a current Drug Enforcement Agency registration that includes Schedule III authority will be able to prescribe buprenorphine for OUD in their practice if permitted by applicable state law.

Several studies have conducted geographic analysis of buprenorphine providers across the U.S.^7^, ^8^, ^9^, ^10^ Previous research suggested that the availability of MOUDs has been slow to expand, whereas there are geographic areas with significant treatment gaps.^11^^,^^12^ This study evaluates the spatial clusters of buprenorphine providers to identify treatment gaps. The study uses CDC mortality data to identify counties needing buprenorphine providers to assess the importance of the treatment gap. This analysis may also be considered to establish a baseline to spatially evaluate the X-waiver elimination and its impact on increasing geographic accessibility for buprenorphine providers and prescriptions in the future.

## METHODS

### Study Population

SAMHSA served as the leading national authority on granting qualifying practitioner waivers to administer, dispense, and prescribe buprenorphine for treating OUD in a qualified practice setting after submission of a NOI and completion of specialized training. The Buprenorphine Waiver Notification System tracked the number of Drug Addiction Treatment Act–waivered providers nationwide by state and patient limit (30, 100, and 275). A limited set of personally identifiable information was collected from providers, including names, medical license numbers, primary addresses, phone numbers, Drug Enforcement Agency numbers, and state licenses. Data are directly collected from qualified practitioners by submitting a buprenorphine waiver NOI at the SAMHSA website (https://www.samhsa.gov/medication-assisted-treatment/become-buprenorphine-waivered-practitioner), or the data may be downloaded from FindTreatment.gov. The NOI application captured information on the provider's qualifying credentials.

### Measures

The data used for this analysis were the cumulative number of all certified buprenorphine providers till the end of December 2022 obtained from the restricted Filemaker database. The study includes all waivered providers on the internal list maintained by SAMHSA, not just limited to the publicly available data through the buprenorphine practitioner locator or FindTreatment.gov. The population data for the spatial analysis were used from the U.S. Census American Community Survey. The analysis also mapped the data on drug-related mortality obtained from CDC WONDER data for context and discussion.

### Spatial Cluster Analysis

Spatial cluster analysis is applied in many fields, primarily in epidemiology and criminology.^13^ Geographic visualizations such as mapping rates are a form of exploratory analysis that may be efficient for reference, but formal testing of space-based hypotheses is far more effective when planning resource allocation or targeted area-specific policies. Spatial analysis thus has more vital implications than a distribution map. This deterministic nature of the spatial analysis can be used to explore further factors that may be responsible for sustaining the cluster, thus assisting in planning targeted intervention. A spatial cluster may be defined as a “geographically bounded group of occurrences of sufficient size and concentration to be unlikely to have occurred by chance.”^14^ A spatial cluster (core areas) is said to be detected within a defined geographic area and has a disproportionate excess of the events when compared with neighboring areas under study.^15^ Spatial clustering analysis may be applied to raw variables or rates when there are no a priori hypotheses concerning the method.^13^ The authors chose to implement Kulldorff's spatial scan statistic method available in the free software tool called SaTScan.^16^, ^17^, ^18^, ^19^, ^20^ The authors used purely spatial discrete Poisson analysis; the method is described in detail in a series of published literature.^16^^,^^19^^,^^21^, ^22^, ^23^ If geographic units (counties in this case) show higher RR of events (buprenorphine providers), the counties may be more likely to be identified as clusters (Appendix Methodology, available online, provides further details).

## RESULTS

Table 1 shows the changes in certified buprenorphine providers by year. The numbers and percentage of buprenorphine providers have increased over the time frame. Table 1 shows that Doctor of Medicine/Doctor of Osteopathic Medicine has the highest number of certified providers at 8,134 (65.08%) in 2018 and 14,525 (57.87%) in 2022. This was followed by NPs at 3,431 (27.45%) in 2018 and 8,073(32.16%) in 2022. The physician assistants were at 933 (7.46%) in 2018 and 2,368 (9.43%) in 2022.

**Table 1:** Descriptive Distribution of the Certified Buprenorphine Providers by Years

All levels	CNM, *n* (%)	CNS, *n* (%)	CRNA, *n* (%)	MD/DO, *n* (%)	NP, *n* (%)	PA, *n* (%)	Total (%)
2018	0 (0)	1 (0.01)	0 (0)	8,134 (65.08)	3,431 (27.45)	933 (7.46)	12,499 (100)
2019	22 (0.11)	15 (0.07)	1 (0)	13,191 (63.90)	5,790 (28.05)	1,625 (7.87)	20,644 (100)
2020	115 (0.5)	53 (0.23)	12 (0.05)	14,489 (62.61)	6,646 (28.72)	1,827 (7.89)	23,142 (100)
2021	80 (0.36)	38 (0.17)	5 (0.02)	13,078 (59.65)	6,699 (30.56)	2,024 (9.23)	21,924 (100)
2022	91 (0.36)	32 (0.13)	10 (0.04)	14,525 (57.87)	8,073 (32.16)	2,368 (9.43)	25,099 (100)

CNM, Certified Nurse Midwife; CNS, Clinical Nurse Specialist; CRNA, Certified Registered Nurse Anesthetist; MD/DO, Doctor of Medicine/Doctor of Osteopathic Medicine; PA, physician assistant.

**Figure 1:**
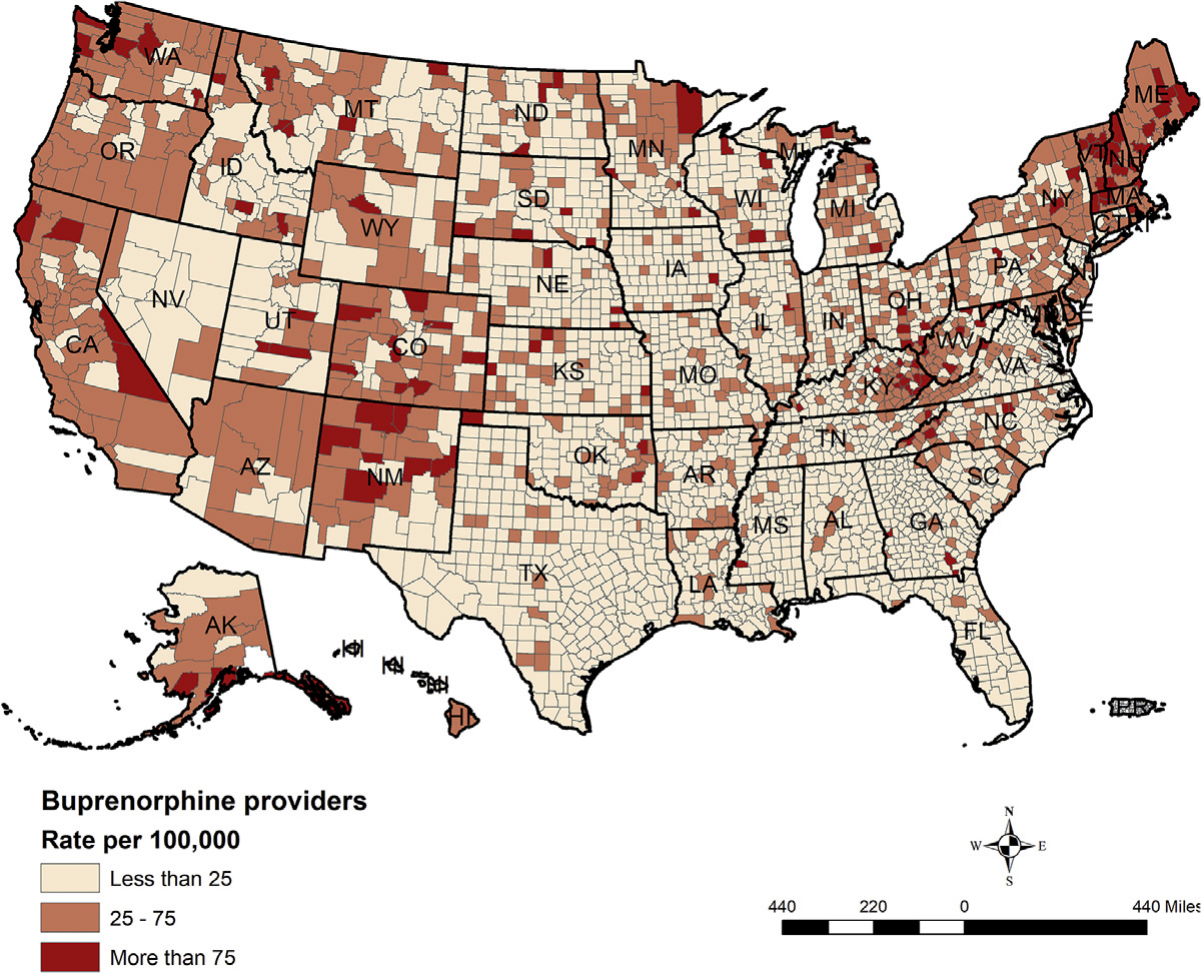
Rate of buprenorphine providers per 100,000 population.

Figure 1 shows the distribution of the rate of providers per 100,000. The figure shows spatial disparity in the distribution across the counties in the U.S. Higher rates of more than 75 providers per 100,000 population were found in the counties of New Mexico, Oregon, Washington, California, and Alaska as well as across the eastern coasts of Maine, Vermont, and New Hampshire. Large parts of the central U.S., such as Indiana and Iowa, have <25 providers per 100,000 population in the county. Figure 1 also shows <25 providers per 100,000 population in the counties of the southern states of Texas, Georgia, and Florida. Appendix Table 2 (available online) shows the number of identified spatial clusters of buprenorphine providers, corresponding *p*-values, and the computed RR for each cluster.

**Figure 2:**
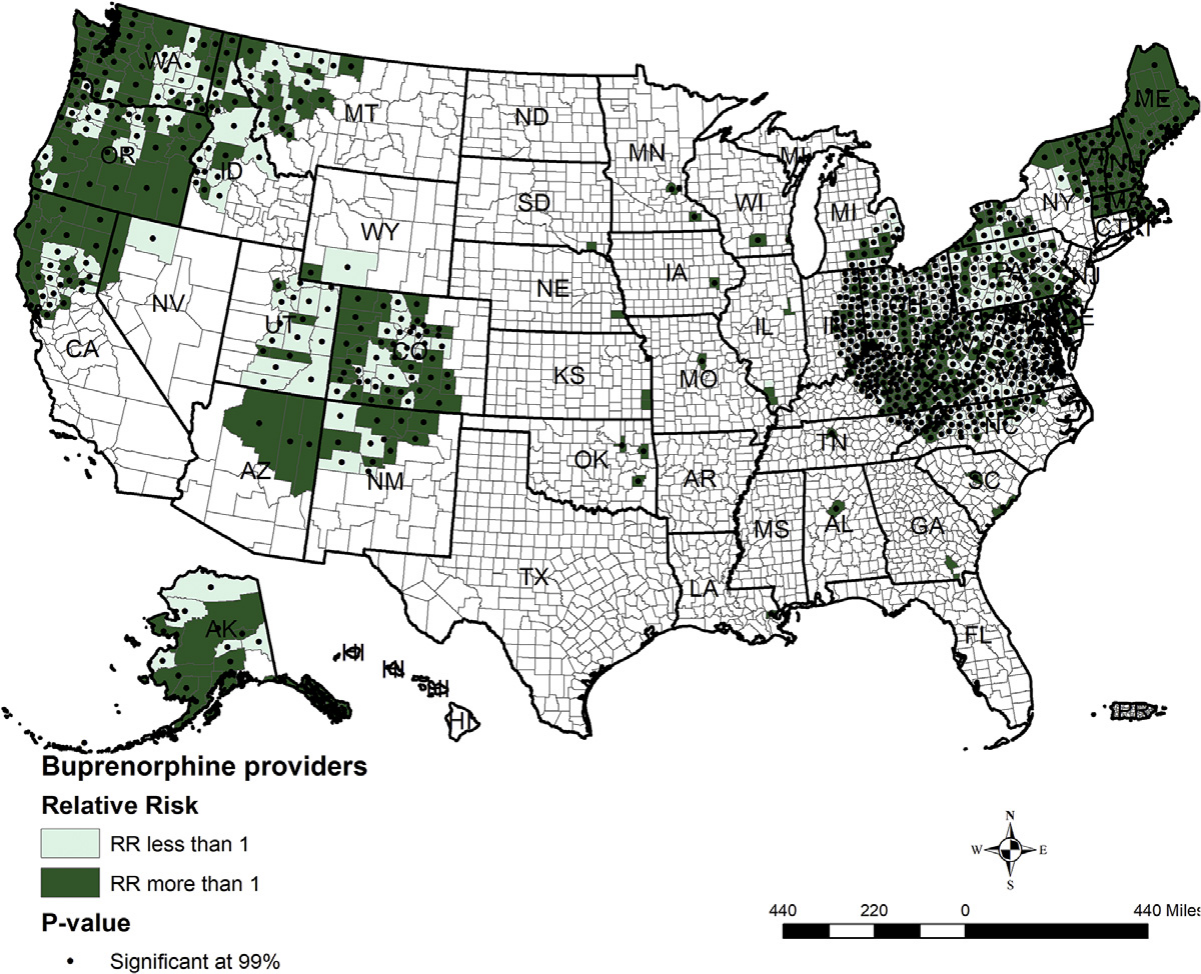
Spatial clusters of certified buprenorphine providers. RR, relative risk.

Figure 2 shows the spatial clusters of buprenorphine providers across the counties of the U.S. The darker green counties with darker dots are counties with RR of more than 1, and corresponding *p*<0.001 were found across the counties in Colorado, northeastern counties of Arizona, and northern New Mexico. Counties with RR of more than 1 and corresponding *p*<0.001 were also evident in the counties of states Washington, Oregon, and Northern California. On the eastern side of the U.S., counties with RR >1 with corresponding *p*<0.001 were found in the northeastern states of Maine, Vermont, and New Hampshire. Few counties in Ohio, Kentucky, and West Virginia have buprenorphine providers with >1 RR and corresponding *p*<0.001. For interpretation, a county with an RR >1 and *p*<0.001 will be considered a statistically significant cluster showing an excessive number of events (in this case, buprenorphine providers) compared with its neighboring areas. Counties in Alaska showed high clusters of buprenorphine providers with an RR >1 and a *p*<0.001.

Appendix Figure 1 (available online) shows the distribution of CDC drug-related mortality rates across counties in the U.S. In this analysis, the counties with high drug-related mortality are considered as primary counties in need of buprenorphine providers. The counties without any color have data either suppressed or not reported. The counties with the darkest colors have higher rates of drug-related mortality and the highest need for buprenorphine providers and access to buprenorphine medications. The counties in West Virginia, New Mexico, Tennessee, and California have drug-related mortality rates of more than 6 people per 100,000.

## DISCUSSION

The summary table in the analysis showed that the total number of buprenorphine providers had increased yearly since 2018. The summary table also showed that the percentage of NPs who could prescribe buprenorphine rose from 2018 to 2022, whereas the rate of Doctor of Medicine/Doctor of Osteopathic Medicine prescribing buprenorphine decreased marginally from years 2018 to 2022.

The analysis demonstrated that buprenorphine providers in the U.S. are clustered. The clusters were evident primarily in the northern counties of the U.S., both on the east and west coasts. The clusters were also found in the counties around the quadripoint of the states in the southwestern U.S. The spatial clusters with higher and significant RRs were found in the counties identified as geographic areas of need on the basis of the drug-related mortality rates. Although higher buprenorphine providers were clustered in areas of need, there are significant gaps of buprenorphine providers in the U.S., which may lead to reduced access to buprenorphine.

On December 22, 2022, the U.S. House of Representatives passed the Restoring Hope for Mental Health and Well-Being Act of 2022 (H.R. 7666), which comprised the Mainstreaming Addiction Treatment Act. The legislation eliminated clinicians’ need to apply for an X-waiver to prescribe MOUD. Under the new law, clinicians must complete 8 hours of training but do not need to apply for a separate waiver. This improved access to treatment by removing a major barrier for those previously ineligible for X-waivers, regardless of education. The elimination is expected to increase the number of physicians prescribing buprenorphine, especially in areas where treatment gaps were evident from the analysis.

The analysis is also expected to assist SAMHSA as the administration continues to address gaps in treatment through expanded programs through funding such as Medication-Assisted Treatment – Prescription Drug and Opioid and Addiction and State Opioid Response. Over the last few years, as the lead agency under HHS, SAMHSA has been working to reduce barriers to access to treat OUD through expansion of funding, implementing educational and outreach programs, implementing HHS priorities-based guidelines to treat OUD, and redesigning FindTreatment.gov (the tool to find substance use and mental health treatment).

### Limitations

Although a crucial contribution to the existing scientific knowledge on MOUD, the analysis suffers from some limitations. The buprenorphine data are a database with limited scope restricted to space and time variables along with provider type. The database collects information that allows a deep dive into the assessment of the study, such as demographic characteristics. Considering that the data are programmatic, it does not allow any scope for ecologic analysis. The database changed constantly as providers got certified, leading to constant changes in the number of providers certified. The overdose mortality data used in the study were pulled from CDC WONDER. The map of overdose-related mortality has suppressed numbers in the counties as well as state reporting of drug-related overdose varies across states. Although the results of this study show the expansion of buprenorphine providers in counties affected by overdose-related mortality, these results are restricted by suppressed counties and nonreporting. Future research studies will require valid and credible sources of data. Still, the reality is that there are currently only private paid sources of data, which may not be ideal for an appropriate study of this phenomenon, given the potential for conflicts of interest in those data sources. SAMHSA's Treatment Episode Data Set may be used to assess the overall expansion of MOUD but not buprenorphine specifically.

## CONCLUSIONS

In this analysis, the authors assessed the expansion of buprenorphine to treat OUD, which is the first medication to treat OUD that can be prescribed or dispensed in physician offices. Buprenorphine offers several advantages to those with OUD and to others for whom treatment in a methadone clinic is not appropriate or is less convenient. Areas with limited access to care and high mortality rates likely represent most incredible opportunity to save lives by increasing treatment options. Armed with geospatial information about regional imbalances between treatment programs and deaths, local and federal authorities can initiate policies targeting geographic areas with the greatest need. The HHS continues to work with partner agencies to address barriers to buprenorphine prescription and overdose prevention.

## CRediT authorship contribution statement

**Suparna Das:** Conceptualization, Methodology, Software, Writing – original draft, Visualization, Investigation, Supervision, Writing – review & editing. **Kasimu Muhetaer:** Data curation. **Neeraj Gandotra:** Supervision. **Naomi Tomoyasu:** Writing – review & editing.

## Appendix Supplementary materials

Image, application 1

Image, application 2

Image, application 3

## Footnotes

Supplementary material associated with this article can be found in the online version at doi:10.1016/j.focus.2024.100284.
