# Supplementary material for: Filling the Treatment Gap: Geographic Expansion of Buprenorphine Providers Across the U.S
Source: AJPM Focus. 2024 Oct 16;4(1):100284. doi: 10.1016/j.focus.2024.100284 (PMC11994037; doi:10.1016/j.focus.2024.100284)
Supplement: Supplementary file 1 [file mmc1.docx]

| CLUSTER | P_VALUE_2018 | CLU_OBS | CLU_EXP | CLU_ODE | CLU_RR | CLU_POP |
| --- | --- | --- | --- | --- | --- | --- |
| 1 | 0.000000000000000010 | 4545 | 2507.23 | 1.81 | 2.27 | 66358412 |
| 1 | 0.000000000000000010 | 4545 | 2507.23 | 1.81 | 2.27 | 66358412 |
| 2 | 0.000000000000000010 | 1620 | 575.84 | 2.81 | 3.08 | 15240751 |
| 3 | 0.000000000000000010 | 1341 | 681.70 | 1.97 | 2.08 | 18042456 |
| 4 | 0.000000000000000010 | 196 | 21.78 | 9.00 | 9.13 | 576498 |
| 5 | 0.000000000000000010 | 411 | 113.42 | 3.62 | 3.71 | 3001824 |
| 6 | 0.000000000000000010 | 1111 | 609.90 | 1.82 | 1.90 | 16142039 |
| 7 | 0.000000000000000010 | 530 | 230.85 | 2.30 | 2.35 | 6109985 |
| 8 | 0.000000000000000010 | 657 | 373.40 | 1.76 | 1.80 | 9882651 |
| 9 | 0.000000000000000010 | 359 | 172.53 | 2.08 | 2.11 | 4566344 |
| 9 | 0.000000000000000010 | 359 | 172.53 | 2.08 | 2.11 | 4566344 |
| 10 | 0.000000000000000010 | 64 | 10.26 | 6.24 | 6.27 | 271534 |
| 11 | 0.000000000000000010 | 235 | 108.39 | 2.17 | 2.19 | 2868768 |
| 12 | 0.000000030704117250 | 31 | 5.63 | 5.51 | 5.52 | 148884 |
| 13 | 0.000000113384754700 | 136 | 68.41 | 1.99 | 2.00 | 1810673 |
| 14 | 0.000003978244148000 | 86 | 38.34 | 2.24 | 2.25 | 1014729 |
| 15 | 0.000021679401719490 | 38 | 11.08 | 3.43 | 3.44 | 293310 |
| 16 | 0.001281493777040010 | 57 | 24.86 | 2.29 | 2.30 | 658046 |

Appendix Table 1: The number of identified significant spatial clusters in 2018 and 2022 and their characteristics.

Clusters for 2018

| Clusters for 2022 | |  |  |  |  |  |
| --- | --- | --- | --- | --- | --- | --- |
| CLUSTER_22 | P_VALUE_2022 | CLU_OBS | CLU_EXP | CLU_ODE | CLU_RR | CLU_POP |
| 1 | 0.00000000000000001 | 20233 | 10128.45 | 2.00 | 2.19 | 26300671 |
| 2 | 0.00000000000000001 | 2192 | 297.01 | 7.38 | 7.49 | 771245 |
| 3 | 0.00000000000000001 | 12815 | 6951.84 | 1.84 | 1.94 | 18051947 |
| 4 | 0.00000000000000001 | 2298 | 607.26 | 3.78 | 3.84 | 1576876 |
| 5 | 0.00000000000000001 | 2753 | 867.37 | 3.17 | 3.22 | 2252305 |
| 6 | 0.00000000000000001 | 2645 | 918.49 | 2.88 | 2.92 | 2385068 |
| 7 | 0.00000000000000001 | 1445 | 313.94 | 4.60 | 4.64 | 815201 |
| 8 | 0.00000000000000001 | 1183 | 222.01 | 5.33 | 5.37 | 576498 |
| 9 | 0.00000000000000001 | 1655 | 607.02 | 2.73 | 2.75 | 1576251 |
| 10 | 0.00000000000000001 | 22483 | 18081.31 | 1.24 | 1.30 | 46951991 |
| 11 | 0.00000000000000001 | 6011 | 3797.88 | 1.58 | 1.61 | 9862016 |
| 12 | 0.00000000000000001 | 2600 | 1252.13 | 2.08 | 2.10 | 3251410 |
| 13 | 0.00000000000000001 | 1375 | 533.21 | 2.58 | 2.60 | 1384602 |
| 14 | 0.00000000000000001 | 2246 | 1110.98 | 2.02 | 2.04 | 2884896 |
| 15 | 0.00000000000000001 | 1334 | 561.34 | 2.38 | 2.39 | 1457638 |
| 16 | 0.00000000000000001 | 991 | 361.95 | 2.74 | 2.75 | 939885 |
| 17 | 0.00000000000000001 | 895 | 309.38 | 2.89 | 2.91 | 803377 |
| 18 | 0.00000000000000001 | 1703 | 853.72 | 1.99 | 2.01 | 2216878 |
| 19 | 0.00000000000000001 | 530 | 142.25 | 3.73 | 3.74 | 369390 |
| 20 | 0.00000000000000001 | 1603 | 825.96 | 1.94 | 1.95 | 2144780 |
| 21 | 0.00000000000000001 | 579 | 182.93 | 3.17 | 3.18 | 475010 |
| 22 | 0.00000000000000001 | 1350 | 697.29 | 1.94 | 1.95 | 1810673 |
| 23 | 0.00000000000000001 | 1008 | 476.79 | 2.11 | 2.12 | 1238090 |
| 24 | 0.00000000000000001 | 370 | 104.57 | 3.54 | 3.55 | 271534 |
| 25 | 0.00000000000000001 | 893 | 456.89 | 1.95 | 1.96 | 1186421 |
| 26 | 0.00000000000000001 | 1224 | 733.99 | 1.67 | 1.67 | 1905956 |
| 27 | 0.00000000000000001 | 1755 | 1156.33 | 1.52 | 1.52 | 3002669 |
| 28 | 0.00000000000000001 | 1713 | 1124.97 | 1.52 | 1.53 | 2921223 |
| 29 | 0.00000000000000001 | 1340 | 836.98 | 1.60 | 1.61 | 2173405 |
| 30 | 0.00000000000000001 | 868 | 481.14 | 1.80 | 1.81 | 1249387 |
| 31 | 0.00000000000000001 | 488 | 217.18 | 2.25 | 2.25 | 563951 |
| 32 | 0.00000000000000001 | 1866 | 1269.47 | 1.47 | 1.48 | 3296445 |
| 33 | 0.00000000000000001 | 1340 | 850.76 | 1.58 | 1.58 | 2209182 |
| 34 | 0.00000000000000001 | 301 | 112.95 | 2.66 | 2.67 | 293310 |
| 35 | 0.00000000000000001 | 375 | 159.06 | 2.36 | 2.36 | 413024 |
| 36 | 0.00000000000000001 | 205 | 62.94 | 3.26 | 3.26 | 163436 |
| 37 | 0.00000000000000001 | 678 | 373.97 | 1.81 | 1.82 | 971102 |
| 38 | 0.00000000000000001 | 169 | 47.84 | 3.53 | 3.54 | 124238 |
| 39 | 0.00000000000000001 | 499 | 258.04 | 1.93 | 1.94 | 670050 |
| 40 | 0.00000000000000001 | 635 | 357.40 | 1.78 | 1.78 | 928059 |
| 41 | 0.00000000000000001 | 463 | 253.41 | 1.83 | 1.83 | 658046 |
| 42 | 0.00000000000000001 | 1056 | 720.00 | 1.47 | 1.47 | 1869622 |
| 43 | 0.00000000000000001 | 209 | 87.27 | 2.39 | 2.40 | 226604 |
| 44 | 0.00000000000000001 | 816 | 546.69 | 1.49 | 1.50 | 1419607 |
| 45 | 0.00000000000000001 | 1451 | 1091.18 | 1.33 | 1.33 | 2833476 |
| 46 | 0.00000000000000001 | 183 | 76.71 | 2.39 | 2.39 | 199182 |
| 47 | 0.00000000000000001 | 454 | 271.09 | 1.67 | 1.68 | 703953 |
| 48 | 0.00000000000000001 | 47 | 6.97 | 6.75 | 6.75 | 18087 |
| 49 | 0.00000000000000022 | 1414 | 1082.13 | 1.31 | 1.31 | 2809969 |
| 50 | 0.00000000000037037 | 1485 | 1172.57 | 1.27 | 1.27 | 3044833 |
| 51 | 0.00000000000115885 | 138 | 59.59 | 2.32 | 2.32 | 154748 |
| 52 | 0.00000000000589839 | 404 | 257.18 | 1.57 | 1.57 | 667820 |
| 53 | 0.00000000001178857 | 199 | 103.13 | 1.93 | 1.93 | 267792 |
| 54 | 0.00000000007386780 | 258 | 148.61 | 1.74 | 1.74 | 385898 |
| 55 | 0.00000000017600688 | 475 | 321.41 | 1.48 | 1.48 | 834621 |
| 56 | 0.00000003231433643 | 241 | 145.17 | 1.66 | 1.66 | 376971 |
| 57 | 0.00000037216674498 | 256 | 161.09 | 1.59 | 1.59 | 418307 |
| 58 | 0.00000060513908917 | 364 | 249.18 | 1.46 | 1.46 | 647054 |
| 59 | 0.00000073700369974 | 234 | 145.09 | 1.61 | 1.61 | 376760 |
| 60 | 0.00000224237577773 | 157 | 88.20 | 1.78 | 1.78 | 229018 |
| 61 | 0.00000447293615657 | 133 | 71.57 | 1.86 | 1.86 | 185840 |
| 62 | 0.00008736654922414 | 62 | 26.08 | 2.38 | 2.38 | 67729 |
| 63 | 0.00018788811039006 | 15 | 2.04 | 7.34 | 7.34 | 5309 |
| 64 | 0.00018978315345508 | 28 | 7.31 | 3.83 | 3.83 | 18970 |
| 65 | 0.00027531923004576 | 48 | 18.34 | 2.62 | 2.62 | 47627 |
| 66 | 0.00029316480600283 | 64 | 28.36 | 2.26 | 2.26 | 73643 |
