# Supplementary material for: Filling the Treatment Gap: Geographic Expansion of Buprenorphine Providers Across the U.S
Source: AJPM Focus. 2024 Oct 16;4(1):100284. doi: 10.1016/j.focus.2024.100284 (PMC11994037; doi:10.1016/j.focus.2024.100284)
Supplement: Supplementary file 2 [file mmc2.docx]

Methodology Supplement

**SaTScan:**

Kulldorff’s spatial scan statistic method places a circular scanning window at each of the point locations within the analysis area. At each of these point locations, we used upper limits of 50% of the population for our analysis, which means that relative risk is calculated if the excess risk is more than 50 percent for this analysis. We chose this based on a trial-and-error method varying the percent but yielded a *p-value < 0.001*. For interpretation a county with relative risk more than 1 and p-value <0.001 will be considered a statistically significant cluster showing excessive number of events (in this case buprenorphine providers) compared to its neighboring areas. This spatial statistical methodology can identify the spatial clusters but should not be used to comment on the reasons as to why the clusters exist without implementing other analysis if feasible and if data is available. Through this process, the method produces many distinct circular windows, each including a different set of neighboring points for the clustering test. The windows that have a significantly higher clustering of events are ‘spatial clusters. The null hypothesis of Kulldorff’s spatial scan statistic states that the event is randomly distributed in geographic space and that the expected event count is proportional to the population at risk ^13^. For the discrete Poisson analysis, a case (buprenorphine provider) and a population (total population in the county) file were created with the tracts as the geographic unit. We used a coordinate file containing the latitude and longitude at the centroid of the county to define the locations for both the analysis ^29^. In this study, the space scan statistic identified the locations and durations of the most likely clusters. Space scan statistics can be used for cluster detection, but it only uses case data and cannot distinguish whether the cluster is due to an increased risk of disease or a sudden increase in population ^27^. Thus, the discrete Poisson model, as used in this analysis, utilizes the extra population information. The population data was obtained from American Community Survey 2021 estimates.

A spatial cluster is said to be detected within a defined geographical area and has a disproportionate excess of the events when compared with neighboring areas under study. An unusual rise or reduction in cases in a specific spatial area can be characterized by statistical significance. The sets of potential clusters are then rank- ordered according to the magnitude of their likelihood ratio test statistics. Once the null hypothesis is rejected and clusters are formed, this means that the number of events detected within the cluster is significantly different from those outside it. The *p-values* were automatically adjusted for these boundary effects. The relative risk (RR) is calculated based on events inside the cluster and outside the cluster where RR greater than 1 means the cluster has a concentration of more events than outside the cluster, the concurrent *p-values* evaluates if the cluster is statistically significant or not ^20,30–32^. Under the null hypothesis, the number of expected cases is proportional to the population size, while the alternative hypothesis requires that the rate of cases is higher within the window than outside. If regions show higher RR of events (buprenorphine providers), the county might be more likely to be identified as clusters. In mathematical notation the RR is estimated as

$$RR= \frac{\frac{c}{E[c)}}{\frac{(C-c)}{(E\left[ C \right]-E\left[ c \right])}}= \frac{\frac{c}{E[c)}}{\frac{(C-c)}{(C-E\left[ c \right])}}$$

Where c is the number of observed cases within the cluster and C is the total number of cases in the data set. Note that the analysis is conditioned on the total number of cases observed E[C] = C. Based on the method we considered those tracts with RR more than 1 and concurrent *p-values* <0.001 to be spatial clusters (also known as core areas).
