# Supplementary figures and images for: Filling the Treatment Gap: Geographic Expansion of Buprenorphine Providers Across the U.S
Source: AJPM Focus. 2024 Oct 16;4(1):100284. doi: 10.1016/j.focus.2024.100284 (PMC11994037; doi:10.1016/j.focus.2024.100284)

Appendix Figure 1: Drug-related crude death rates (CDR) in 2021 across U.S. counties


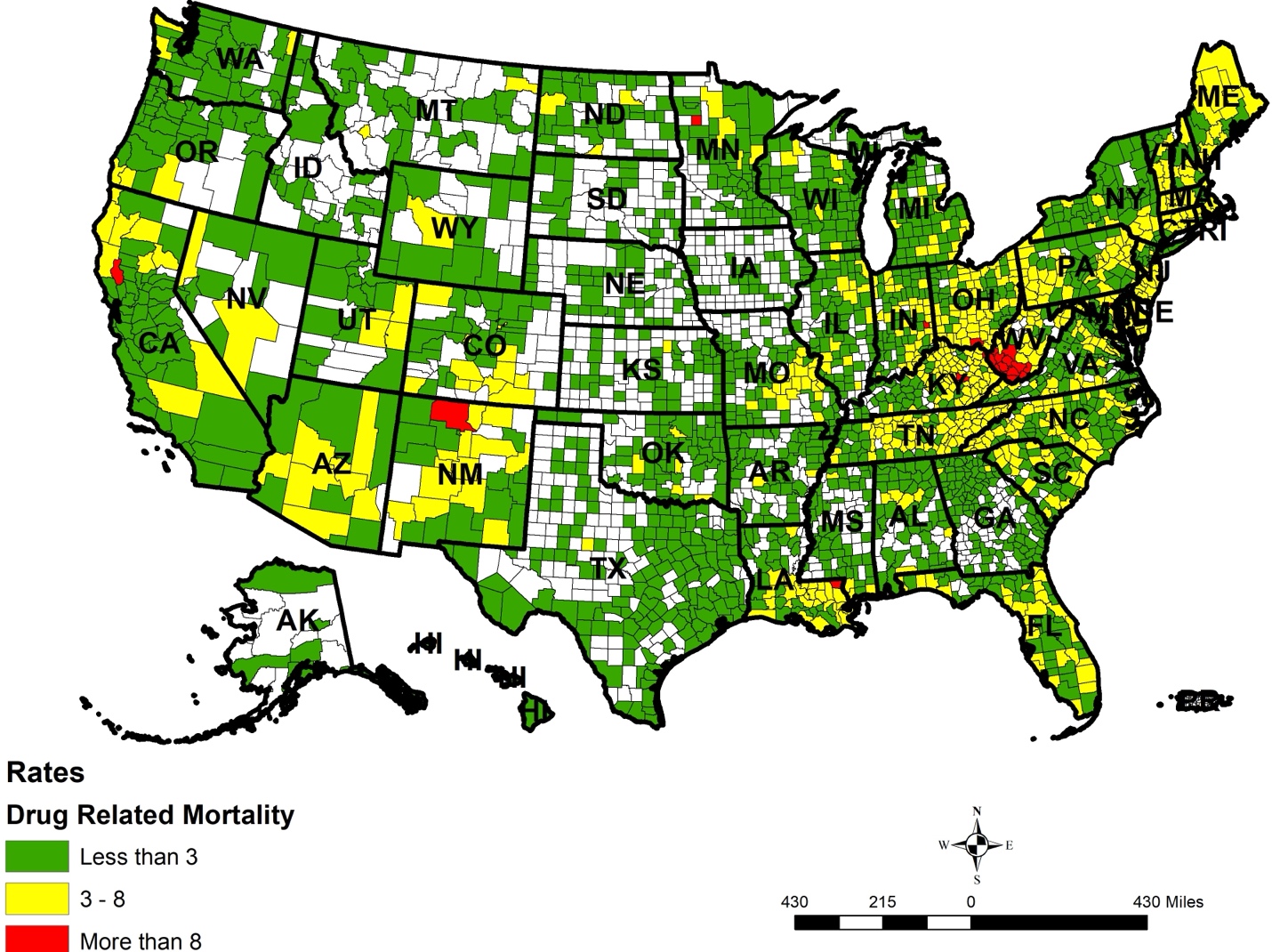

Supplement: Supplementary file 3 [file mmc3.docx]
